# Effects of different exercise interventions on body composition in women with overweight and obesity: a systematic review and network meta-analysis

**DOI:** 10.1136/bmjopen-2025-113206

**Published:** 2026-06-17

**Authors:** Shiwei Song, Yincheng Wei, Haoze Zhang, Andrew Soundy

**Affiliations:** 1 School of Sport, Exercise and Rehabilitation Sciences, University of Birmingham, Birmingham, UK; 2 School of Strength and Conditioning Training, Beijing Sport University, Beijing, China

**Keywords:** Obesity, Exercise, Systematic Review

## Abstract

**Abstract:**

**Objective:**

This study aimed to investigate the effects of different types of exercise on body composition in women with overweight or obesity and to compare the relative effectiveness of these interventions using a systematic review and network meta-analysis.

**Design:**

Systematic review and network meta-analysis using the CINeMA (Confidence in Network Meta-Analysis) approach.

**Data sources:**

PubMed, Embase, CINAHL, SportsDiscus, the Cochrane Library, Web of Science and Scopus were searched through September 2025.

**Eligibility criteria for selecting studies:**

We included randomised controlled trials enrolling adult women with overweight or obesity that evaluated structured exercise interventions, including aerobic exercise, resistance training, aerobic exercise with resistance training, high-intensity interval training or whole-body vibration training. Eligible studies compared these interventions with control conditions or other exercise modalities and reported at least one body composition outcome, including body fat percentage, body mass index, fat mass, lean body mass or waist circumference.

**Data extraction and synthesis:**

Two independent reviewers used standardised methods to search, screen and code included studies. Risk of bias was assessed using the Cochrane Collaboration and Evidence Project tools. Network meta-analysis was conducted using random effects models. Confidence in the estimates was assessed using the CINeMA approach.

**Results:**

43 randomised controlled trials involving 2315 women were included. For body fat percentage, aerobic exercise with resistance training, high-intensity interval training and aerobic exercise across varying intensities were associated with greater reductions relative to control conditions, with vigorous aerobic exercise showing the highest probability of benefit. A similar pattern was observed for other adiposity-related outcomes: vigorous aerobic exercise appeared most favourable for body mass index, high-intensity interval training for fat mass and moderate to vigorous intensity aerobic exercise for waist circumference. No intervention was associated with statistically significant improvements in lean body mass compared with control; however, resistance training demonstrated a comparatively favourable ranking profile.

**Conclusions:**

Structured exercise confers meaningful improvements in adiposity among women, particularly with aerobic-based modalities, whereas effects on lean mass remain limited. These results underscore the importance of tailored exercise strategies for optimising body composition in female populations.

**PROSPERO registration number:**

CRD420251161064.

**STRENGTHS AND LIMITATIONS OF THIS STUDY**

- This study employed a comprehensive network meta-analysis, allowing for both direct and indirect comparisons across seven distinct exercise modalities, enhancing the robustness of comparative effectiveness estimates.
- The literature search spanned seven major databases and included grey literature sources, citation chasing and search engine screening, minimising the risk of publication bias and maximising study capture.
- Risk of bias was rigorously assessed using the Cochrane tool and certainty of evidence was evaluated via the Confidence in Network Meta-Analysis framework, ensuring transparent appraisal of study quality and confidence in findings.
- The retrospective registration of the International Retrospective Register of Systematic Reviews protocol may introduce concerns regarding protocol adherence and transparency, although this was addressed and clarified in the revised manuscript.
- Despite tailoring search terms to database-specific indexing systems, variability in terminology and indexing across studies may have led to missed eligible trials or inconsistent classification of exercise intensity.

## Introduction

 Overweight and obesity among women have become pressing global public health concerns, given their strong association with a range of chronic conditions, including cardiovascular diseases.^1^,^5^ According to data from the WHO, the prevalence of obesity among women has steadily increased over the past decades.^6 7^ Although both classifications are derived from body mass index (BMI), they represent clinically distinct and non-overlapping populations. Evidence suggests that the association between BMI and adverse health outcomes may be non-linear, with obesity generally conferring a greater cardiometabolic risk burden than overweight status.^2^ Obesity in women is associated not only with elevated risks of cardiovascular disease, type 2 diabetes and metabolic syndrome but also with adverse effects on reproductive and mental health.^8^ Nevertheless, excess adiposity constitutes a shared underlying pathophysiological feature across the spectrum of elevated BMI, which provides a common target for lifestyle-based interventions.^1^ Optimisation of body composition including reductions in BMI, body fat percentage, waist circumference and fat mass, together with the preservation of lean body mass, is regarded as a key target for improving obesity-related health outcomes.^9^ As a non-pharmacological and cost-effective strategy, exercise interventions have been widely implemented in weight management among overweight and obese populations.^10^

Previous research has demonstrated that different types of exercise exert varying effects on body composition. For instance, aerobic exercise has been shown to enhance energy expenditure and fat oxidation, thereby contributing to reductions in body weight and body fat percentage.^11^ Alternatively, resistance training increases muscle mass, elevates basal metabolic rate and prevents the loss of lean body mass during weight loss.^12^ High-intensity interval training has attracted increasing attention due to its time efficiency and pronounced metabolic effects.^13^ Moreover, combined exercise modalities, such as aerobic and resistance training, may provide greater benefits by simultaneously reducing fat mass and preserving lean body mass.^14^

However, despite the growing number of randomised controlled trials (RCTs) investigating exercise interventions in overweight and obese populations, most existing meta-analyses have been limited to traditional pairwise comparisons. These analyses typically focus on direct comparisons between a few commonly studied modalities, such as aerobic exercise, resistance training, combined aerobic and resistance training, whole-body vibration training and high-intensity interval training. However, they often fail to account for the broader landscape of available interventions. While such pairwise approaches have yielded valuable insights into the efficacy of individual exercise types, their scope remains inherently constrained. They are unable to incorporate indirect evidence from trials that do not share a common comparator, and they lack the capacity to generate a comprehensive ranking of intervention effectiveness.

For instance, Campa *et al*^15^ investigated the effects of different resistance training frequencies on body composition in overweight and obese women, reporting improvements in lean mass and reductions in fat mass, but without comparing resistance training to other modalities such as high-intensity interval training or aerobic exercise. Liu *et al*^16^ synthesised evidence on various resistance exercise forms, yet their pairwise meta-analysis excluded indirect comparisons and lacked a unified ranking framework.^16^ Amare *et al*^17^ compared aerobic exercise, resistance training and aerobic exercise with resistance training in terms of fat mass and glucolipid metabolism, but did not include emerging modalities like whole-body vibration training or high-intensity interval training. Jayedi *et al*^18^ conducted a dose-response meta-analysis of aerobic exercise, confirming linear reductions in body fat and waist circumference with increasing aerobic exercise duration, yet did not assess comparative effectiveness across modalities.^18^

These fragmented findings highlight the limitations of pairwise meta-analytic approaches in synthesising the full spectrum of exercise interventions. Without a unified analytical framework that accommodates both direct and indirect comparisons, it remains difficult to determine which exercise modality offers the greatest benefit for improving body composition outcomes.

Importantly, network meta-analysis (NMA) enables the integration of direct and indirect evidence, the ranking of multiple interventions and the generation of more comprehensive evidence to inform clinical decision-making.^19^ The validity of NMA relies on the assumption of transitivity, which requires comparability across studies in terms of population characteristics, intervention contexts and outcome definitions. Although overweight and obesity are clinically distinct BMI categories, exercise-induced effects on body composition are primarily mediated through shared mechanisms related to energy balance, substrate utilisation and skeletal muscle adaptation. In the present study, inclusion was restricted to adult women with elevated BMI, and outcome measures were consistently defined, thereby enhancing the plausibility of the transitivity assumption.

Therefore, this study was designed to systematically evaluate the effects of different types of exercise on body composition in overweight or obese women using NMA, with the aim of identifying the most effective exercise strategies.

## Methods

### Registration

This systematic review and NMA were reported in accordance with the Preferred Reporting Items for Systematic Reviews and Meta-Analyses (PRISMA-NMA) guidelines,^20^ and the search reported according to the PRISMA-S statement for reporting literature searches.^21^ The study protocol was registered in the International Retrospective Register of Systematic Reviews (PROSPERO; ID: CRD420251161064). This was a retrospective registration due to the study being part of a larger research initiative and oversight during project planning; it was subsequently registered once the scope was finalised. See online supplemental file 1 for completed PRISMA diagram.

### Patient and public involvement

No public and patient involvement activities were undertaken for this research.

### Literature search strategy

Two authors conducted the electronic search strategy. Two reviewers independently screened titles and abstracts and subsequently full-text articles for eligibility. Any disagreements were resolved through discussion, with a third reviewer available for arbitration.

The search strategy was developed according to the core elements of the PICOS framework: (P) Population: women with overweight or obesity; (I) Intervention: exercise; (C) Comparator: no exercise or normal-lifestyle condition; (O) Outcomes: the primary outcome was body fat percentage, with secondary outcomes including lean body mass, BMI, waist circumference and fat mass; and (S) Study design: RCTs only.

A total of seven electronic databases were searched from inception to September 2025. Articles were retrieved from PubMed, Embase, CINAHL, SportsDiscus, the Cochrane Library, Web of Science and Scopus following a systematic electronic search. For searches conducted in PubMed/the Cochrane Library and Embase, MeSH and Emtree terms were used, respectively. Keywords used for searching included terms (“adipose tissue hyperplasia” OR adipositas OR adiposity OR “alimentary obesity” OR “body weight, excess” OR corpulency OR “fat overload” OR “syndrome nutritional” OR obesity OR obesitas OR overweight) AND (females OR woman OR women OR female) AND (Exercises OR “Exercise, Physical” OR “Exercises, Physical” OR “Physical Exercise” OR “Physical Exercises” OR “Exercise, Aerobic” OR “Aerobic Exercise” OR “Aerobic Exercises” OR “Exercises, Aerobic” OR “Exercise Training” OR “Exercise Trainings” OR “Training, Exercise” OR “Trainings, Exercise” OR “Physical Activity” OR “Activities, Physical” OR “Activity, Physical” OR “Physical Activities” OR “Tai Chi” OR “resistance training” OR “strength training” OR “combined training” OR Qigong OR “Whole-Body Vibration Training” OR Baduanjin) AND (“body component” OR “lean body mass” OR “body weight” OR “body adiposity index” OR “body fat” OR “body fat percentage”) AND (“randomized controlled trial” OR randomized OR placebo). Detailed search strategies for each database are provided in online supplemental file S2.

In addition, two search engines were searched for the first 30 pages of results including Google Scholar and ScienceDirect. Grey literature was searched using ProQuest and GreyMatters. Citation chasing was performed on all included articles. See online supplemental file S2 for completed search strategies of electronic databases.

### Eligibility criteria

Studies were included if they met the following criteria, defined according to the PICOS framework, with an additional section for other criteria.

Population (P): Participants were classified as overweight or obese according to regional BMI cut-offs when available. For European populations, overweight was defined as BMI (25.0–29.9 kg/m²) and obesity as BMI ≥30 kg/m². For Asian populations, overweight was defined as BMI ≥24 kg/m² and obesity as BMI ≥28 kg/m². For studies conducted in other regions or when regional cut-offs were not specified, the BMI classification criteria of the WHO (overweight: BMI ≥25 kg/m²; obesity: BMI ≥30 kg/m²) were applied.

If BMI data were unavailable, body fat percentage was used as an alternative criterion (female body fat percentage ≥30%). Eligible participants were adult women aged 18–65 years. For studies including women older than 65 years, data had to be reported separately, and the older subgroup could not exceed 30% of the total sample to minimise the potential confounding effects of age on body composition. Studies combining exercise with other interventions (eg, caloric restriction, pharmacological interventions) were excluded unless both exercise-only and control groups without caloric restriction were reported. In addition, animal studies were excluded.

Intervention (I): The intervention group received at least 8 weeks of structured exercise training. For the purpose of this review, exercise was generally defined as physical activity that is structured, planned and repetitive and has a goal of improvement or maintenance of an individual’s physical fitness.^22^ Subclassification of exercise acceptable for this review is identified in table 1. Studies were excluded if they reported only the acute effects of a single exercise session.

**Table 1:** The classifications of exercise training

Type	Definition
Control	No structured exercise intervention or continuation of normal lifestyle without prescribed physical activity.
Vigorous-intensity aerobic exercise	Type: Continuous aerobic exercise only (eg, walking, running, cycling). Intensity: >65% VO_2max,_ >65% HRR or >75% MHR.^24^
Moderate-to-vigorous intensity aerobic exercise	Type: Continuous aerobic exercise only. Intensity: Exercise intensity spanning moderate and vigorous ranges, typically progressing from moderate to vigorous intensity over the intervention period.^24^
Moderate-intensity aerobic exercise	Type: Continuous aerobic exercise only. Intensity: 45%–65% VO_2max,_ 50%–65% HRR or 65%–75% MHR.^24^
Resistance training (RT)	Type: Structured resistance exercise targeting major muscle groups using free weights, machines or resistance bands; includes traditional and circuit-based programmes. Intensity: ≥50% one-repetition maximum (1RM).^22 24^
Aerobic exercise combined with RT	Type: Programmes incorporating both aerobic exercise and RT within the same intervention period, either within the same session or on separate days. Intensity prescribed according to aerobic and RT criteria above.^24^
High-intensity interval training	Type: Exercise involving repeated short-to-long bouts of high-intensity aerobic effort interspersed with recovery periods; includes sprint interval training. Intensity: >65% VO_₂max_, >65% HRR or >75% MHR during high-intensity intervals.^60 61^
Whole-body vibration training	Type: Exercise performed on a vibrating platform while maintaining static or dynamic postures or performing movements. Mechanical parameters: Typically delivered at frequencies between ~20 and 50 Hz with low-to-moderate amplitude.^48^

HRR, heart rate reserve; MHR, maximum heart rate; RM, repetition maximum; VO_2max_, maximal oxygen uptake.

Comparator (C): Studies required an active or inactive control group. The control group could involve no exercise or normal-lifestyle condition.

Outcomes (O): Studies had to assess changes in body fat percentage before and after the intervention, with no restriction on measurement methods.

Study design (S): Only RCTs were included. Studies were excluded if they were qualitative in nature, a literature review, editorial letters or abstracts from conference proceedings.

Other criteria: Studies were excluded if they were duplicate publications. No date restriction was placed on studies. Only English language RCTs published up to September 2025 were included.

Two researchers independently screened all retrieved studies according to the predefined inclusion and exclusion criteria. A third reviewer was available for arbitration if required. Multiple reports from the same trial were consolidated, with the first or most comprehensive report selected as the primary reference.

### Exercise categories

Given the heterogeneity of exercise interventions, classification required careful consideration. Categories were informed by established exercise science frameworks^23^ and were operationalised during full-text review and data extraction. Interventions were grouped according to their primary modality, intensity characteristics and physiological emphasis. Although categories were finalised after reviewing included studies, they were based on conceptual criteria rather than outcome data, and no restructuring was performed following network estimation.

Eight categories were used to classify the exercise interventions for the included RCTs (table 1). Vigorous exercise intensity is defined as a maximal oxygen uptake (VO2max) >65%, heart rate reserve (HRR) >65% or maximum heart rate (MHR) >75%, while moderate exercise corresponds to 45%–65% VO2max, 50%–65% HRR or >65%–75% MHR.^24^

### Data extraction

Data were independently extracted by two researchers, and any discrepancies were resolved through discussion, with a third author consulted if necessary. Extracted information included the first author, year of publication, country, participant characteristics (number of participants in experimental and control groups, sex, age, body fat percentage, BMI, lean body mass, fat mass and waist circumference), intervention details (type of exercise, intensity, duration, frequency, intervention period and whether supervised or unsupervised), as well as outcome measurement methods and units. When data were incomplete, corresponding authors were contacted via email to obtain the missing information.

### Risk of bias and Confidence in Network Meta-Analysis assessment

The risk of bias 1.0 (ROB 1.0) of included studies was independently assessed by two researchers using the Cochrane Risk of Bias Assessment Tool,^25^ which evaluates seven domains: (a) random sequence generation, (b) allocation concealment, (c) blinding of participants and personnel, (d) blinding of outcome assessment, (e) incomplete outcome data, (f) selective reporting and (g) other sources of bias. Because participant blinding is difficult to implement in exercise interventions, this domain was excluded from the overall risk of bias scoring; instead, the blinding of personnel in outcome assessment was used as a quality criterion. An overall ROB assessment was identified as used in previous NMA. Studies with no high-risk domains and no more than three unclear-risk domains were classified as low risk; studies with at least one high-risk domain, or with four or more unclear-risk domains in the absence of high-risk domains, were classified as moderate risk; all remaining studies were categorised as high risk.^23 26^ The certainty of evidence for primary and secondary outcome network estimates was assessed using the Confidence in Network Meta-Analysis (CINeMA) framework, which encompasses recommendation grading, assessment, formulation and evaluation.^27^

### Outcomes

The primary outcome of interest was body fat percentage. Secondary outcomes included BMI, waist circumference, fat mass and lean body mass. Given that body composition outcomes were assessed using different measurement methods and were occasionally reported using different units or scales, standardised mean differences (SMDs) were calculated to enable comparison on a common scale across studies.

For studies reporting outcomes at multiple time points, the measurement taken closest to the end of the intervention period was selected, as prespecified in the protocol. When multiple eligible timepoints were available within the same window, the timepoint most commonly reported across studies was chosen to maximise consistency. All standardisation and selection procedures were defined a priori.

### Data synthesis and statistical analyses

The preintervention and postintervention change values for the experimental and control groups were combined to estimate the effect. The SD of the changes was calculated according to the formula provided in the Cochrane Handbook for Systematic Reviews of Interventions (V.6.3).^28^

A random-effects multivariable non-parametric meta-analysis was performed using STATA V.16.0 (StataCorp).^29^ Following the frequentist framework and current PRISMA guidelines for non-parametric meta-analyses, pooled estimates and 95% CIs were calculated.^20^ Owing to differences in outcome measurement tools across studies, SMDs were used to assess the effect sizes of body fat percentage, BMI, fat mass, lean body mass and waist circumference.

Multiarm trials were incorporated using the built-in multiarm adjustment of the frequentist NMA model implemented in STATA (network suite; mvmeta). When a study included multiple intervention arms sharing a common comparator, the model accounted for the within-trial correlation by estimating the appropriate variance-covariance structure. This approach prevents double-counting of shared control groups and avoids inflation of study weights or underestimation of standard errors. No sample-size splitting or post-hoc adjustments were required, as multiarm structures were handled directly within the NMA framework.

The relationships between exercise interventions were illustrated using a network evidence diagram, in which the lines connecting nodes represent direct comparisons between interventions, and the size of each node and the thickness of connecting lines are proportional to the number of studies. A network contribution plot was also generated to quantify the contribution of each direct comparison.

The transitivity assumption of NMA was evaluated by examining the inclusion criteria of individual studies, assessing whether all participants could theoretically be randomised to any intervention and applying a consistency model.^30^ Transitivity, a core assumption of NMA, assumes that indirect comparisons can reliably reflect unobserved direct comparisons when effect modifiers are evenly distributed across studies.^29 31^ To evaluate the plausibility of the transitivity assumption, baseline BMI distributions across treatment comparisons were examined descriptively to assess potential imbalances (see online supplemental file S3). Consistency within each closed-loop was assessed by calculating inconsistency factors with 95% CIs, with consistency indicated when the lower limit of the 95% CI included 0.^32^ An inconsistency model was applied to test for overall inconsistency. When inconsistency was not significant (p>0.05), a consistency model was used.^33^ Local inconsistency was further evaluated using node-splitting analysis, and results were considered reliable when p>0.05. The Surface Under the Cumulative Ranking curve (SUCRA) was used to summarise the relative ranking probabilities of different exercise interventions.^34^ SUCRA values range from 0 to 100, with higher values indicating a greater probability of an intervention ranking closer to the top of the hierarchy. However, SUCRA does not directly reflect the magnitude of effect, clinical relevance, or certainty of evidence, and rankings should therefore be interpreted cautiously.^35^ To assess potential publication bias due to small-study effects, a network funnel plot was constructed and visually inspected for symmetry.^36^

## Results

### Literature selection

**Figure 1:**
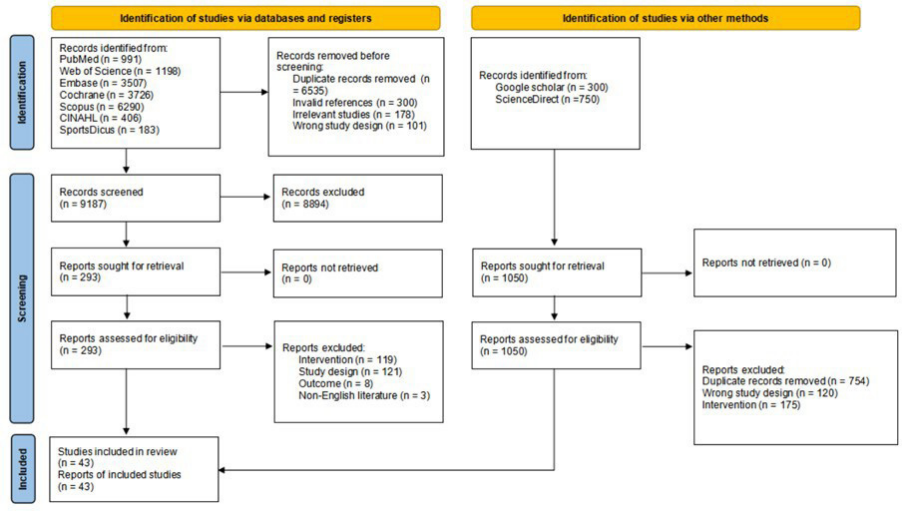
Preferred Reporting Items for Systematic Reviews and Meta-Analyses (PRISMA) flow diagram depicting the study selection process.

Following PRISMA guidelines, 17 351 records were identified through database searches and 1050 through additional sources. After removal of 6535 duplicates and preliminary exclusions, 9187 records underwent title and abstract screening, with 8894 excluded. Full texts of 293 articles were assessed for eligibility, and 250 were excluded due to intervention mismatch, inappropriate study design, outcome inconsistency or language restrictions. Records identified from other sources were further excluded for duplication, design ineligibility or intervention mismatch. Ultimately, 44 studies met the predefined inclusion criteria and were included in the systematic review (figure 1).

### Characteristics of the included studies

The characteristics of the included studies are summarised in online supplemental file S3, with a complete list provided in online supplemental file S4. These studies were published between 2006 and 2025 and conducted across North America (4/43, 9%), South America (3/43, 7%), Asia (23/43, 51%), Europe (13/43, 30%) and Africa (1 study, 2%). The experimental groups comprised a total of 1599 female participants with overweight or obesity, whereas the control groups included 716 participants. Participant ages ranged from 18 to 65 years. Baseline characteristics included age, BMI, body fat percentage, fat mass and lean body mass.

Exercise interventions were categorised as moderate aerobic exercise, moderate to vigorous aerobic exercise, vigorous aerobic exercise, resistance training, aerobic exercise with resistance training, high-intensity interval training and whole-body vibration training. Several studies applied progressive overload principles, which limited the ability to clearly differentiate training intensity between resistance training and aerobic exercise with resistance training groups. The durations reported in online supplemental file S3 excluded warm-up and cool-down sessions unless otherwise specified. On average, the intervention period was 12.6 weeks, ranging from 8 to 40 weeks, with over one-sixth (6/43, 14%) of studies lasting longer than 12 weeks. Participants trained an average of 3.3 sessions per week, and all interventions were delivered under supervised conditions.

Regarding outcome assessment, body fat percentage was measured using multiple methods, including Bioelectrical Impedance Analysis, dual-energy X-ray absorptiometry (DEXA), skinfold thickness with Siri/Jackson formulas and hydrostatic weighing. Bioelectrical Impedance Analysis devices from InBody, TANITA and Omron Healthcare, as well as the electronic scale Ohaus, were employed across studies. Secondary outcome measures included BMI, lean body mass, fat mass and waist circumference. See online supplemental file S3 for characteristics of included studies and online supplemental file S4 for the list of included studies.

### Results of ROB assessment

Details of the ROB 1.0 assessment for each study are provided in online supplemental file S5. A total of 29 (29/43, 67%) studies explicitly reported their randomisation methods. Regarding allocation procedures, 19 (19/43, 44%) studies demonstrated evidence of allocation concealment, and 22 (22/43, 51%) studies reported the use of blinding in outcome assessment. Additionally, 40 (40/43, 93%) studies were judged to have a low risk of selective reporting. For other sources of bias, studies were rated as high risk when they involved small sample sizes (fewer than 10 participants per group), lacked supervision mechanisms or exhibited substantial measurement error in outcome assessment. Overall, 28 (28/43, 65%) studies were assessed as having a low risk of bias, 12 (12/43, 28%) as having a moderate risk of bias and 3 (3/43, 7%) as having a high risk of bias.

### Network meta-analysis

An NMA of body fat percentage as the primary outcome was conducted, along with secondary outcomes including lean body mass, BMI, waist circumference and fat mass. This analysis was designed to examine whether the effects of different exercise modalities on secondary outcomes were consistent with changes in body fat percentage, thereby providing a more comprehensive evaluation of intervention effectiveness. In addition, subgroup analyses were conducted based on publication year, assessment methods and intervention duration to assess their potential impact on treatment effects and network heterogeneity.

Supporting materials for body fat percentage and secondary outcomes are presented as follows.

**Figure 2:**
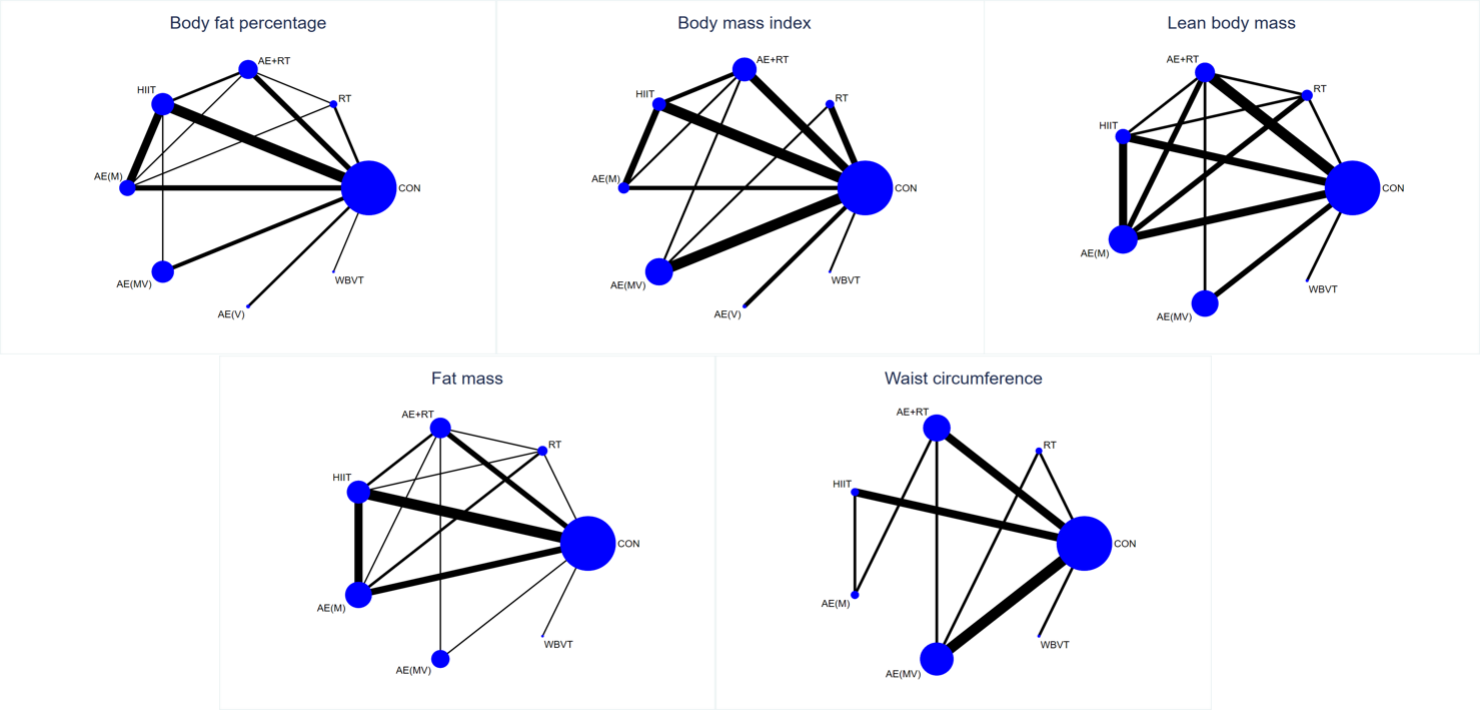
Network plot presenting the effects of different exercise types on body fat percentage and secondary outcomes in women with overweight and obesity. AE, aerobic exercise; RT, resistance training; CON, control; HIIT, high-intensity interval training; and WBVT, whole-body vibration training. Values of exercise intensity are identified in brackets; M, moderate; MV, moderate to vigourous, and V, Vigorous.

Figure 2 presents the network evidence diagram illustrating the NMA of included studies on the effects of exercise types on body fat percentage and secondary outcomes (lean body mass, BMI, waist circumference and fat mass). Node size represents the sample size for each exercise modality, while the thickness of connecting lines indicates the number of studies comparing the respective interventions. Moderate intensity aerobic exercise (12/43, 28%) was the most frequently investigated intervention, whereas whole-body vibration training (1/43, 2%) was the least common.

Online supplemental file S6 presents the network contribution plot, illustrating the contributions of direct and indirect comparisons to the NMA and the number of studies informing each direct comparison.

Inconsistency between body fat percentage and secondary outcomes was assessed using loop-specific heterogeneity estimates, inconsistency models and node-splitting analysis (see online supplemental file S7). The loop-specific approach showed good consistency for all closed loops in body fat percentage and BMI, while three loops in lean body mass, three in FM and one in waist circumference exhibited inconsistency. The inconsistency model yielded p values >0.05 for lean body mass, waist circumference and fat mass, indicating no significant inconsistency. In addition, inclusion of two body fat percentage studies and one BMI study resulted in statistically significant global inconsistency (p<0.05), as assessed by the design-by-treatment interaction model. Sensitivity analyses indicated that these studies substantially affected network consistency; therefore, they were excluded from the final model (see online supplemental file S7).^37^,^39^ Node-splitting analysis further confirmed that, overall, there was no inconsistency between direct and indirect evidence, supporting the reliability of the results.

Forest plots for eligible comparisons of body fat percentage and secondary outcomes, including 95% CIs and 95% prediction intervals (95% PrIs), are presented in online supplemental file S8.

Funnel plots for body fat percentage and secondary outcomes were constructed to assess potential publication bias in the NMA (see online supplemental file S9). The plots were approximately symmetrical for all outcomes, suggesting a low likelihood of publication bias or small-study effects.

The SUCRA probabilities for each intervention in the network, for body fat percentage and secondary outcomes, are presented in online supplemental file S10. Higher SUCRA values indicate a greater probability that an intervention ranks closer to the top of the hierarchy within the network, rather than reflecting the magnitude or certainty of its effect.

### Pooled estimates of primary outcomes

Tables2  3 present the results of the league table analysis for body fat percentage. Compared with the control group, aerobic exercise with resistance training (SMD=−0.86, 95% CI −1.36 to −0.36), high intensity interval training (SMD=−0.88, 95% CI −1.28 to −0.49), moderate aerobic exercise (SMD = −0.78, 95% CI −1.28 to −0.29), moderate to vigorous aerobic exercise (SMD=−0.88, 95% CI −1.45 to −0.31) and vigorous aerobic exercise (SMD=−1.15, 95% CI −2.09 to −0.21) were associated with significant reductions in bodyfat percentage.

**Table 2:** A network meta-analysis matrix of body fat percentage and secondary outcome measures

Body fat percentage							
RT							
0.60 (−0.16,1.37)	AE+RT						
0.63 (−0.12,1.38)	0.03 (−0.51,0.57)	HIIT					
0.53 (−0.24,1.30)	−0.08 (−0.69,0.54)	−0.10 (−0.57,0.37)	AE(M)				
0.62 (−0.26,1.51)	0.02 (−0.72,0.76)	−0.01 (−0.65,0.63)	0.09 (−0.64,0.82)	AE(MV)			
0.90 (−0.27,2.06)	0.29 (−0.77,1.36)	0.27 (−0.75,1.29)	0.37 (−0.70,1.43)	0.27 (−0.83,1.37)	AE(V)		
0.52 (−0.95,1.99)	−0.09 (−1.48,1.31)	−0.11 (−1.47,1.25)	−0.01 (−1.40,1.38)	−0.10 (−1.52,1.32)	−0.38 (−1.98,1.23)	WBVT	
−0.25 (−0.94,0.43)	**−0.86 (−1.36,−0.36**)	**−0.88 (−1.28,−0.49**)	**−0.78 (−1.28,−0.29**)	**−0.88 (−1.45,−0.31**)	**−1.15 (−2.09,−0.21**)	−0.77 (−2.07,0.53)	CON
Body mass index							
RT							
0.66 (−0.26,1.57)	AE+RT						
0.60 (−0.33,1.52)	−0.06 (−0.69,0.56)	HIIT					
0.48 (−0.56,1.52)	−0.18 (−0.93,0.58)	−0.12 (−0.82,0.59)	AE(M)				
0.29 (−0.56,1.14)	−0.37 (−1.07,0.33)	−0.31 (−1.06,0.44)	−0.19 (−1.07,0.69)	AE(MV)			
**1.44** (**0.08,2.80**)	0.78 (−0.45,2.02)	0.85 (−0.41,2.10)	0.96 (−0.38,2.30)	1.15 (−0.10,2.40)	AE(V)		
0.48 (−1.14,2.10)	−0.18 (−1.72,1.36)	−0.12 (−1.66,1.43)	−0.00 (−1.61,1.61)	0.19 (−1.35,1.73)	−0.96 (−2.79,0.87)	WBVT	
−0.16 (−0.91,0.59)	**−0.82 (−1.36,−0.28**)	**−0.75 (−1.31,−0.20**)	−0.64 (−1.36,0.08)	**−0.45** (**−0.98,0.09**)	**−1.60 (−2.73,−0.47**)	−0.64 (−2.08,0.80)	CON
Lean body mass							
RT							
−0.35 (−1.26,0.57)	AE+RT						
−0.38 (−1.30,0.54)	−0.04 (−0.81,0.73)	HIIT					
−0.49 (−1.35,0.37)	−0.14 (−0.88,0.60)	−0.10 (−0.80,0.60)	AE(M)				
−0.01 (−1.17,1.15)	0.33 (−0.57,1.24)	0.37 (−0.66,1.40)	0.48 (−0.54,1.49)	AE(MV)			
0.09 (−1.67,1.84)	0.43 (−1.22,2.08)	0.47 (−1.20,2.14)	0.57 (−1.09,2.24)	0.10 (−1.64,1.83)	AE(V)	WBVT	
−0.04 (-0.91,0.83)	0.30 (-0.32,0.92)	0.34 (−0.33,1.01)	0.44 (−0.22,1.10)	−0.03 (−0.85,0.79)	—	−0.13 (−1.66,1.40)	CON
Fat mass							
RT							
**0.81** (**0.10,1.51**)	AE+RT						
**0.86** (**0.20,1.52**)	0.05 (−0.45,0.56)	HIIT					
0.39 (−0.25,1.04)	−0.41 (−0.97,0.14)	**−0.47 (−0.90,−0.03**)	AE(M)				
0.72 (−0.23,1.68)	−0.08 (−0.85,0.68)	−0.14 (−0.95,0.68)	0.33 (−0.51,1.17)	AE(MV)			
0.60 (−0.81,2.00)	−0.21 (−1.55,1.12)	−0.26 (−1.57,1.04)	0.20 (−1.12,1.52)	−0.13 (−1.59,1.33)	AE(V)	WBVT	
−0.09 (−0.73,0.54)	**−0.90 (−1.35,−0.44**)	**−0.95 (−1.33,−0.57**)	**−0.48 (−0.91,−0.06**)	**−0.81 (−1.56,−0.07**)	—	−0.69 (−1.94,0.57)	CON
Waist circumference							
RT							
0.44 (−0.90,1.77)	AE+RT						
0.19 (−1.23,1.61)	−0.25 (−1.19,0.69)	HIIT					
0.07 (−1.55,1.68)	−0.37 (−1.42,0.67)	−0.12 (−1.24,1.00)	AE(M)				
0.56 (−0.65,1.76)	0.12 (−0.68,0.91)	0.37 (−0.61,1.34)	0.49 (−0.73,1.71)	AE(MV)			
0.65 (−1.25,2.55)	0.21 (−1.40,1.82)	0.46 (−1.20,2.12)	0.58 (−1.25,2.42)	0.09 (−1.50,1.69)	AE(V)	WBVT	
−0.19 (−1.39,1.01)	−0.62 (−1.27,0.02)	−0.37 (−1.14,0.40)	−0.25 (−1.35,0.85)	**−0.74 (−1.36,−0.12**)	—	−0.84 (−2.31,0.63)	CON

Bold value indicates that the longitudinal intervention has a more significant reduction impact than the horizontal intervention.

AE, aerobic exercise; AE(M), moderate-intensity AE; HIIT, high-intensity interval training; MV, moderate to vigorous intensity; RT, resistance training; V, vigorous-intensity; WBVT, whole-body vibration training.

**Table 3:** Ranking of exercise interventions in order of effectiveness

Body fat percentage		Body mass index		Lean body mass		Fat mass		Waist circumference	
Treatment	SUCRA	Treatment	SUCRA	Treatment	SUCRA	Treatment	SUCRA	Treatment	SUCRA
CON	5.1	CON	9.0	CON	65.5	CON	9.4	CON	17.30
RT	19.5	RT	22.6	RT	65.3	RT	16.7	RT	36.50
AE+RT	61.6	AE+RT	67.5	AE+RT	35.1	AE+RT	75.8	AE+RT	65.90
HIIT	64.5	HIIT	62.6	HIIT	32.8	HIIT	80.6	HIIT	46.90
AE(M)	54.4	AE(M)	53.0	AE(M)	23.5	AE(M)	42.8	AE(M)	39.20
AE(MV)	62.9	AE(MV)	40.5	AE(MV)	64.0	AE(MV)	67.3	AE(MV)	73.80
AE(V)	77.2	AE(V)	92.8	AE(V)	—	AE(V)	—	AE(V)	—
WBVT	54.6	WBVT	52.0	WBVT	63.9	WBVT	57.5	WBVT	70.30

AE, aerobic exercise; BFP, body fat percentage; CON, control; FM, fat mass; HIIT, high-intensity interval training; LBM, lean body mass; M, moderate intensity; MV, moderate to vigorous intensity; RT, resistance exercise; SUCRA, Surface Under the Cumulative Ranking curve; V, vigorous intensity; WBVT, whole-body vibration training; WC, waist circumference.

According to SUCRA values, vigorous aerobic exercise was relatively higher for reducing body fat percentage (77.2), followed by high intensity interval training (64.5), moderate to vigorous aerobic exercise (62.9), aerobic exercise with resistance training (61.6), whole body vibration training (54.6) and moderate aerobic exercise (54.4), whereas resistance training ranked relatively lower (19.5). These rankings reflect the relative probability of each intervention within the network rather than definitive evidence of superiority. Neither resistance training nor whole body vibration training demonstrated statistically significant effects on body fat percentage compared with the control group or other exercise modalities.

### Pooled estimates of the secondary outcome

We also performed an NMA on secondary outcomes, including lean body mass, BMI, waist circumference and fat mass, to determine whether the effects of different exercise modalities on these outcomes were consistent with their effects on body fat percentage.

An NMA was performed to compare the effects of different exercise modalities on body composition outcomes.

For BMI, the following results were identified: aerobic exercise with resistance training (SMD=−0.82, 95% CI −1.36 to −0.36), high intensity interval training (SMD=−0.75, 95% CI −1.31 to −0.20), moderate intensity aerobic exercise (SMD=−0.64, 95% CI −1.36 to −0.08) and vigorous aerobic exercise (SMD=−1.60, 95% CI −2.73 to −0.47) were associated with significant reductions compared with the control group. Additionally, vigorous aerobic exercise demonstrated relatively greater reductions compared with resistance training (SMD=1.44, 95% CI 0.08 to 2.80). Based on SUCRA values, vigorous intensity aerobic exercise ranked relatively higher (92.8), followed by aerobic exercise with resistance exercise (67.5), high intensity interval training (62.6), moderate intensity aerobic exercise (53.0), whole-body vibration training (52.0) and moderate intensity aerobic exercise (40.5), whereas resistance training ranked relatively lower (22.6). No other statistically significant pairwise differences were observed.

For fat mass, the following results were identified: aerobic exercise with resistance training (SMD=−0.90, 95% CI −1.35 to −0.44), high intensity interval training (SMD=−0.95, 95% CI −1.33 to −0.57), moderate aerobic exercise (SMD=−0.48, 95% CI −0.91 to −0.06) and moderate to vigorous aerobic exercise (SMD=−0.81, 95% CI −1.56 to −0.07) were associated with significant reductions compared with control. Moreover, aerobic exercise with resistance training (SMD=0.81, 95% CI 0.10 to 1.51) and high intensity interval training (SMD=0.86, 95% CI 0.20 to 1.52) demonstrated relatively greater reductions than resistance training alone, and high intensity interval training showed relatively greater effects than moderate aerobic exercise (SMD=−0.47, 95% CI −0.90 to −0.03). According to SUCRA values, high intensity interval training ranked relatively higher (80.6), followed by aerobic exercise with resistance training (75.8), moderate intensity aerobic exercise (67.3) and whole-body vibration training (57.5), while resistance training ranked relatively lower (16.7).

For lean body mass, the following results were observed: none of the exercise modalities demonstrated statistically significant improvements compared with control. SUCRA values suggested a possible ranking pattern, with resistance training (65.3), moderate intensity aerobic exercise (64.0) and whole-body vibration training (63.9) ranking relatively higher than other modalities. However, given the absence of statistically significant differences, these rankings should be interpreted cautiously.

Regarding waist circumference, the following results were observed; moderate to vigorous aerobic training was the only intervention associated with a significant reduction compared with control (SMD=−0.74, 95% CI −1.36 to −0.12). SUCRA rankings indicated that moderate to vigorous aerobic exercise ranked relatively higher (73.8), followed by whole-body vibration training (70.3), resistance training and aerobic exercise (65.9), high intensity interval training (46.9), moderate intensity aerobic exercise (39.2) and resistance training (36.5). See online supplemental file S11 for forest plots of individual studies.

### Subgroup NMA of primary outcome

A subgroup analysis of body fat percentage was performed based on publication year, assessment method and intervention duration to explore whether these covariates influenced the study outcomes (see online supplemental file S14). The corresponding subgroup network diagram is presented in online supplemental file S12.

Analysis of subgroup-specific heterogeneity demonstrated good consistency across all closed loops for recent studies (≤5 years), while five loops in recent studies (>5 years), four in DEXA and six in Bioelectrical Impedance Analysis exhibited inconsistency. The inconsistency model indicated no significant inconsistency for publication year and assessment method (p>0.05). In contrast, the subgroup stratified by intervention duration failed to meet the consistency assumption, implying potential disagreement between direct and indirect evidence. Node-splitting analysis further indicated that the direct and indirect comparison results were consistent across all subgroups.

In the subgroup of recent studies, the NMA for body fat percentage showed that aerobic exercise with resistance training (SMD=−1.24, 95% CI −2.47 to −0.02) and high intensity interval training (SMD=−1.02, 95% CI −1.83 to −0.21) were associated with significant reductions in body fat percentage compared with the control group. Based on SUCRA rankings, aerobic exercise with resistance training (SUCRA=69.7) ranked relatively higher among the interventions, whereas resistance (SUCRA=26.2) showed a lower ranking probability.

In the earlier studies subgroup, significant reductions in body fat percentage were observed for aerobic exercise with resistance training (SMD=−0.79, 95% CI −1.35 to −0.22), high intensity interval training (SMD=−0.79, 95% CI −1.31 to −0.27), AE(M) (SMD=−0.89, 95% CI −1.59 to −0.19) and moderate to vigorous intensity aerobic exercise (SMD=−0.86, 95% CI −1.47 to −0.26) compared with controls. SUCRA analysis indicated that moderate intensity aerobic exercise (SUCRA=71.4) ranked relatively higher, while resistance training (SUCRA=28.0) again demonstrated a lower ranking probability.

When stratified by assessment method, the bioelectrical impedance analysis subgroup demonstrated comparatively larger effect sizes, with aerobic exercise with resistance training (SMD=−2.19, 95% CI −4.20 to −0.18) and high intensity interval training (SMD=−2.76, 95% CI −4.85 to −0.66) showing significant reductions in body fat percentage. According to SUCRA rankings, high intensity interval training (SUCRA=85.7) appeared more favourable relative to other interventions, whereas resistance training (SUCRA=19.4) ranked lower.

In contrast, within the DEXA subgroup, aerobic exercise with resistance training (SMD=−0.69, 95% CI −1.16 to −0.22), high intensity interval training (SMD=−0.67, 95% CI −1.07 to −0.26), moderate intensity aerobic exercise (SMD=−0.56, 95% CI −1.05 to −0.07) and moderate intensity aerobic exercise (SMD=−0.75, 95% CI −1.43 to −0.06) were all associated with significant reductions in body fat percentage. SUCRA rankings suggested that moderate to vigorous intensity aerobic exercise (SUCRA=72.3) ranked relatively higher among the included interventions, while resistance training (SUCRA=29.3) consistently showed a lower ranking probability.

### CINeMA assessment

Online supplemental file S13 presents the CINeMA assessments for each comparison, together with the SUCRA-based rankings for the primary and secondary outcomes. Overall, the certainty of evidence for most comparisons involving body fat percentage, lean body mass, fat mass, BMI and waist circumference was rated as moderate to low, indicating that the SUCRA rankings should be interpreted with appropriate caution.

## Discussion

Building on prior research, this study provides a critical refinement and expansion of the evidence base by specifically investigating the effects of exercise in women with overweight and obesity and by incorporating a broader spectrum of exercise modalities. This systematic review and NMA included 43 RCTs involving 2315 female participants. The analysis revealed a distinct, outcome-specific hierarchy of effectiveness: Vigorous intensity aerobic exercise demonstrated the most favourable profile for BMI and body fat percentage reduction, high intensity interval training for fat mass reduction, and moderate to vigorous intensity aerobic exercise for waist circumference reduction, whereas no modality showed definitive superiority for improving lean body mass. While ranking patterns varied across subgroups, aerobic exercise, resistance training and high-intensity interval training consistently demonstrated relatively favourable profiles, whereas resistance training alone generally ranked lower. However, variations across publication periods and assessment methods likely reflect methodological heterogeneity rather than true differences in efficacy.

Restricting the analysis to women represents a deliberate methodological strategy aimed at reducing clinical heterogeneity and improving internal validity. Gender-specific physiological differences are well documented and may substantially influence exercise-induced adaptations in body composition.^40 41^ Compared with men, women typically exhibit higher relative body fat, lower absolute lean mass, distinct regional fat distribution, and a hormonal milieu characterised by fluctuations in oestrogen and progesterone.^40 42^ These biological differences are known to influence substrate utilisation patterns and lipid oxidation during exercise, as well as muscle protein synthesis and recovery responses to resistance training.^43^,^45^ Pooling male and female participants within a single network could therefore violate the transitivity assumption by introducing sex as a strong effect modifier. By focusing exclusively on female populations, the present study provides more targeted evidence to inform exercise prescription for women.

However, this design choice necessarily limits generalisability. The findings should not be directly extrapolated to male populations, as sex differences in fat loss dynamics, hypertrophic response and metabolic adaptation to exercise have been consistently reported.^40 41^ Future research incorporating sex-stratified network models or direct comparisons between men and women would help determine whether the relative ranking of exercise modalities is preserved across sexes.

Variability in the reporting and implementation of exercise intensity and modality across primary studies represents a potential source of misclassification. Although predefined criteria were applied to categorise interventions, differences in training prescription, supervision and reporting detail may have led to some overlap between resistance training and combined aerobic plus resistance training interventions. Such non-differential misclassification would most likely attenuate true between-group differences, thereby biasing estimates towards the null rather than artificially inflating treatment effects. While this may have influenced the magnitude of effect estimates or the precision of SUCRA-based rankings to some extent, it is unlikely to have fundamentally altered the overall pattern of relative effectiveness observed across interventions. Nevertheless, the findings should be interpreted with consideration of the inherent variability in exercise reporting among clinical trials.

### Effect of exercise on primary outcomes

Our findings suggest that structured exercise may contribute to reductions in body fat percentage. Favourable effects were observed across several modalities, including aerobic training and resistance exercise, high-intensity interval training, moderate intensity, moderate to vigorous intensity and vigorous intensity aerobic exercise, although the magnitude of benefit varied.

Previous meta-analyses have consistently demonstrated that aerobic exercise is effective in improving body composition among individuals with overweight and obesity, particularly through reductions in body fat percentage.^46^ However, by synthesising a broader and more recent body of evidence in the present study, we observed that the efficacy of aerobic exercise may be influenced by exercise intensity. Specifically, our NMA revealed that vigorous intensity aerobic exercise demonstrated a significant effect in reducing body fat percentage and achieved the highest SUCRA score, while both moderate intensity aerobic exercise and moderate to vigorous intensity aerobic exercise showed lower effect sizes and SUCRA values compared with vigorous intensity aerobic exercise, suggesting that vigorous intensity aerobic exercise may have a relatively higher probability of being among the more effective interventions. This aligns with recent findings suggesting a dose-and-response relationship between exercise intensity and adiposity outcomes. For instance, Khodadadi *et al*^47^ reported that high-intensity interventions yielded greater reductions in body fat percentage compared with moderate or low-intensity protocols. These findings underscore the importance of tailoring exercise prescriptions not only by modality but also by intensity, to maximise improvements in body composition.

Contrary to earlier meta-analytic findings suggesting that resistance training may contribute to reductions in body fat percentage,^48^ our results did not identify a statistically significant effect of resistance training on body fat percentage, and resistance training showed the relatively lower SUCRA value among all interventions. This divergence may stem from considerable heterogeneity in resistance training protocols across studies, including variations in exercise modality, total training volume, repetition schemes and inter-set work-to-rest ratios. Such variability complicates quantitative synthesis and may obscure potential effects. compared with aerobic exercise, resistance training interventions exhibit greater inconsistency in design and implementation, which likely contributes to the mixed outcomes observed. For instance, Liu *et al*^16^ noted that different resistance training formats (eg, circuit and traditional) yielded divergent effects on body composition in overweight populations. Therefore, resistance training alone may be less consistently associated with reductions in body fat percentage in the present analysis, although it may offer complementary benefits when combined with other modalities.

The present findings reinforce existing evidence regarding the efficacy of aerobic exercise with resistance training in improving body fat percentage among individuals with overweight and obesity.^47^ Our NMA showed that aerobic exercise and resistance training significantly reduced body fat percentage in women and ranked as the fourth most effective intervention according to SUCRA probability estimates. This is consistent with prior meta-analyses indicating that multimodal exercise regimens yield superior outcomes in body composition compared with single-modality interventions.^49 50^ Resistance training, while primarily recognised for its role in enhancing muscular strength and preserving lean mass, may exert synergistic effects when combined with aerobic exercise, contributing to reductions in adiposity and improvements in muscle and bone density.^17 51^ Collectively, these findings suggest that integrating aerobic exercise and resistance training may be beneficial when designing exercise programmes aimed at comprehensive improvements in body composition.

High intensity interval training could significantly improve body fat percentage with a moderate effect in individuals with overweight and obesity compared with controls. The SUCRA probability ranking showed that high intensity interval training ranked only second to vigorous intensity aerobic exercise, indicating a relatively high probability of favourable performance within the network. These findings suggest that high intensity interval training may represent a time-efficient strategy for decreasing body fat percentage. We found that vigorous intensity aerobic exercise and high intensity interval training were among the more effective types of exercise to reduce body fat percentage, and the common feature of both exercises was high-intensity stimulation, and a shared characteristic between them was the application of high-intensity stimuli. The application of high-intensity stimuli may partly explain their relatively greater effects on adiposity reduction.^52 53^

The findings of this study regarding whole body vibration training do align with previous research, as whole body vibration training did not significantly improve body fat percentage.^48^ This discrepancy may be attributed to the limited number of included studies and the stricter inclusion criteria applied in the present analysis, which only considered interventions lasting 8 weeks or longer, whereas most existing studies implemented protocols of approximately 6 weeks.

### Secondary outcomes

The analysis of secondary outcomes suggests that the relative effectiveness of exercise interventions varies according to the specific outcome assessed, highlighting the multifaceted nature of body composition changes.

With respect to both BMI and fat mass, multiple exercise modalities were associated with significant reductions compared with control. However, the pattern of between-intervention differences varied across outcomes. For BMI, although vigorous intensity exercise, aerobic exercise with resistance training, high intensity interval training and moderate intensity aerobic exercise demonstrated significant improvements relative to control, vigorous intensity aerobic exercise ranked relatively higher according to SUCRA probability, the only statistically significant difference between active interventions was observed between vigorous aerobic exercise and resistance training. This finding is consistent with previous meta-analyses indicating that aerobic-based interventions generally produce modest but significant reductions in body weight among individuals with overweight and obesity, whereas resistance training alone tends to exert smaller effects on total body mass.^36 54^ These data suggest that, for BMI, most structured exercise approaches yield broadly comparable benefits, with limited separation between modalities beyond the relatively weaker performance of resistance training alone.

In contrast, a clearer hierarchy emerged for fat mass. Both aerobic exercise with resistance training and high intensity interval training demonstrated greater reductions than resistance training alone, and high intensity interval training also showed a modest advantage over moderate-intensity aerobic exercise. These findings align with prior evidence suggesting that higher-intensity aerobic exercise and combined training strategies may induce greater reductions in adiposity compared with moderate-intensity or single-modality resistance programmes.^55 56^ Mechanistically, higher-intensity exercise may elicit greater total energy expenditure, enhanced post-exercise oxygen consumption and improved metabolic flexibility, while combined aerobic exercise with resistance training protocols may promote complementary adaptations in substrate utilisation and muscle mass that facilitate fat loss. The greater discrimination observed for fat mass compared with BMI in the present analysis may reflect the higher sensitivity of direct adiposity measures to exercise-specific metabolic adaptations, as BMI does not distinguish between fat and lean tissue compartments. Collectively, these findings suggest that while structured exercise in general improves weight-related outcomes, differences in intensity and modality appear more consequential for reducing fat mass than for altering BMI.

The findings for lean body mass and waist circumference, however, presented somewhat divergent patterns. No intervention significantly improved lean body mass compared with control, which is noteworthy in the case of resistance training. However, the SUCRA ranking suggested that resistance training had the highest probability of being ranked favourably for lean body mass, indicating a potential signal of effect that may not have reached statistical significance in the direct and indirect comparisons. This underscores that while resistance training effect on absolute lean body mass may appear modest in these studies, it may still play an important role in preserving lean body mass during weight loss, representing a potential advantage over purely aerobic regimens.^12 57^ For waist circumference, a marker of abdominal adiposity, moderate to vigorous intensity aerobic exercise was the only intervention associated with a statistically significant effect, whereas aerobic exercise with resistance training demonstrated the highest SUCRA probability within the network. This discrepancy between statistical significance and SUCRA values for waist circumference warrants cautious interpretation, particularly given the overlapping CIs across interventions. Although combined training may be a promising approach for targeting central obesity, the present findings should not be interpreted as definitive evidence of superiority.

A potential concern relates to the pooling of overweight and obese participants defined using either BMI or body fat percentage, as these measures may act as effect modifiers for exercise-induced changes in body composition. Although both are indicators of adiposity, BMI reflects body mass relative to height without distinguishing fat from lean tissue, whereas body fat percentage provides a more direct estimate of adiposity. Consequently, individuals classified under these criteria may differ in baseline body composition and metabolic profile. In addition, BMI categories themselves are clinically distinct and may further modify intervention effects. However, baseline BMI values across included studies showed substantial overlap, and no systematic imbalance across intervention comparisons was observed. Therefore, while residual effect modification cannot be entirely excluded, a major violation of the transitivity assumption appears unlikely.

### Subgroup NMA of primary outcome

Subgroup analyses revealed variations in the relative performance of exercise modalities according to publication period and assessment method; however, these findings should be interpreted as exploratory.

When stratified by publication period, both earlier and more recent studies demonstrated significant reductions in body fat percentage for aerobic exercise with resistance training and high intensity interval training compared with control. In earlier studies, moderate intensity aerobic exercise and moderate to vigorous intensity aerobic exercise also showed significant effects, whereas in more recent studies statistically significant reductions were primarily observed for aerobic exercise and resistance training and high intensity interval training. Although SUCRA rankings differed between time periods, with moderate intensity aerobic exercise ranking relatively higher in earlier studies and aerobic exercise and resistance training ranking higher in more recent studies, no systematic pattern of superiority was evident in direct comparisons across subgroups. Such variability may reflect differences in study design, intervention intensity, duration, participant characteristics, or analytical approaches rather than true temporal shifts in comparative efficacy.^24 56^ These findings should be considered hypothesis-generating rather than indicative of evolving optimal exercise strategies.

When stratified by body composition assessment method, distinct patterns also emerged. Effect sizes in the Bioelectrical Impedance Analysis subgroup were comparatively larger, particularly for aerobic exercise and resistance training and high intensity interval training, whereas effect estimates in the DEXA subgroup were more modest but consistently significant across several aerobic-based modalities. Given documented differences in precision and sensitivity between these methods, Bioelectrical Impedance Analysis may be more susceptible to short-term fluctuations in hydration and soft tissue distribution, while DEXA is considered a more accurate reference for adiposity estimation.^58 59^ The higher SUCRA ranking for high intensity interval training in the Bioelectrical Impedance Analysis subgroup and for moderate to vigorous intensity aerobic exercise in the DEXA subgroup should therefore be interpreted in light of methodological differences rather than as definitive evidence of modality superiority. Overall, these subgroup findings underscore the influence of study characteristics and measurement techniques on estimated intervention effects and highlight the need for cautious interpretation of comparative rankings within network meta-analytic frameworks.

### Strengths and limitations

This study has several strengths. A comprehensive NMA enabled both direct and indirect comparisons across seven exercise modalities, enhancing the robustness of comparative effectiveness estimates. The search strategy was extensive, covering seven major databases along with grey literature and citation tracking, thereby minimising publication bias. Risk of bias was assessed using the Cochrane tool, and certainty of evidence was evaluated through the CINeMA framework, ensuring transparent appraisal of study quality.

Several limitations should also be acknowledged. Two studies with substantial heterogeneity were excluded during preliminary assessment, which may have slightly reduced representativeness. Variability in reporting of exercise interventions may have led to minor misclassification; for instance, short-term sprint interval training was categorised as high intensity interval training for analytical consistency. In addition, inconsistencies in terminology across databases could have resulted in missed studies. Finally, the limited number and small sample sizes of whole-body vibration training trials restrict the strength of conclusions for this modality.

## Conclusions

This NMA defines a comparative hierarchy of exercise modalities for improving body composition in women. Interventions incorporating aerobic components consistently demonstrated favourable effects on adiposity-related outcomes, with vigorous aerobic exercise showing the highest probability of benefit overall. High intensity interval training and moderate to vigorous aerobic exercise appeared particularly advantageous for fat mass. In contrast, effects on lean body mass were modest across modalities, although RT showed a comparatively favourable profile. In conclusion, these findings highlight the modality-specific and outcome-dependent nature of exercise adaptations and emphasise the importance of targeted, individualised exercise prescription for optimising body composition in female populations.

## Supplementary material

10.1136/bmjopen-2025-113206 online supplemental file 1

## Data Availability

All data relevant to the study are included in the article or uploaded as supplementary information.
