# Supplementary material for: Effects of different exercise interventions on body composition in women with overweight and obesity: a systematic review and network meta-analysis
Source: BMJ Open. 2026 Jun 17;16(6):e113206. doi: 10.1136/bmjopen-2025-113206 (PMC13288685; doi:10.1136/bmjopen-2025-113206)
Supplement: Supplementary file 2 [file bmjopen-2025-113206.reviewer_comments.pdf]

PEER REVIEW HISTORY

BMJ Open publishes all reviews undertaken for accepted manuscripts. Reviewers are asked to complete a checklist review form and are provided with free text boxes to elaborate on their assessment. These free text comments are reproduced below.

ARTICLE DETAILS

Title (Provisional)

Effects of Different Exercise Interventions on Body Composition in Women with Overweight and Obesity: A Systematic Review and Network Meta-analysis

Authors

Song, Shiwei; Wei , Yincheng; Zhang , Haoze; Soundy, Andrew

VERSION 1 - REVIEW

**Reviewer**	**1**
**Name**	**Dack, Kyle**
**Affiliation**	**University of Bristol, MRC Integrative Epidemiology Unit**
**Date**	**03-Jan-2026**
**COI**	**None**

Statistical review summary:

Generally, this review and network meta-analysis is clear, transparent, and applies best statistical practices. The core of the analysis and its interpretation seems reasonable. However, I have a few queries: (a) a few studies were excluded as outliers – this did not seem to be pre-specified and the justification was unclear, (b) obesity and overweight status are two clinically distinct terms, and to assume they are comparable in meta-analysis is a strong assumption which it would be valuable to justify and explore more strongly, and (c) the review included Abassi et al 2023 which was a study of adolescents aged 15-18 – this does not meet the reported inclusion criteria of participants aged 18-65. The results also reported a minimum participant age of 11, which again should not be possible.

Abstract:

1. I suggest reporting all searched databases and the RoB tool to align with PRISMA.

Introduction:

2. While obese and overweight status are both categories derived from the same measurement BMI), these are clinically distinct terms, based upon the same measurement BMI, Overweight and obese status are both based upon BMI, but are non-overlapping populations. There is evidence of heterogeneity in terms of health outcomes (e.g. a non-

linear effect). The introduction does not currently differentiate the two terms, or explain the rationale for pooling them.

-The NMA assumes transitivity – comparable population/intervention/study design – to enable valid indirect effects, so both from an interpretation point of view and a statistical perspective it is important to establish that these are comparable populations.

Methods:

3. "This systematic review and NMA were conducted in accordance" suggest replacing "conducted" with "reported", because PRISMA is only a reporting guideline, not a methods guideline. I recommend also clarifying that it was PRISMA-NMA, not the standard PRISMA.

4. Search strategy: I suggest reporting the PICOS prior to the specific search terms, because the latter is determined based on the former.

5. "Population (P): participants were overweight and/or obese, defined as BMI > 25 kg/m² (overweight) and/or BMI ≥ 30 kg/m² (obese) for European populations" With the current phrasing, the "and/or" is redundant because BMI > 30 necessarily implies a BMI > 25. It may help to add an upper bound for overweight - I believe the NHS defines overweight status as having a BMI of 25-29.9?

6. Exercise categories: It can be challenging to classify heterogenous interventions into a set of categories adequate for NMA. Could the authors add any further details on how these classifications were developed? Also, were these defined before or after the search?

7. Table 1. I am unsure how it would look – but the authors may wish to consider adding an extra "full term" column, where the "type" is fully described. While all the acronyms were defined earlier or in the footnote, this table is essentially serving as a study-specific key, and making it as self-contained and as readable as possible would help readers who are likely to refer back to it.

8. I suggest adding an "outcome" subheading to define the primary and secondary outcomes. There is details of this in PRISMA. This could be used to describe how outcomes were standardised (currently in the data analysis section), and how a single outcome was selected when there were multiple available (e.g. multiple timepoints, what logic was used to consistently select one timepoint).

Data analysis:

9. -There appear to be multi-arm trials included but it is not clear how these were handled – if within-trial correlations are unaccounted for this could inflate the weighting and underestimate SE.

10. -The pooling of obese and overweight participants – these are clinically heterogeneous, and BMI is a potential modifier for the effect of exercise interventions on body composition, and this may threaten the transitivity assumption. If there is adequate data, this may be worth exploring as an effect modifier or in any other way.

11. -Could intervention duration also potentially introduce heterogeneity? There was a minimum length of 8 weeks which helps to address this, but there is still quite a lot of variation (8-40 weeks).

12. -There is a missing reference at the end of this section.

Results:

13. Figure 1. “records marked as ineligible by automation tools” it is not clear what means – recommend adding this to the methods. 279 records were also removed “for any other reason”, and I suggest stating in-text what this commonly involved.

14. Figure 1. The bottom-right box appears to have inaccurate statistics, considering the number of assessed studies was 1.

15. The inclusion criteria stated that participants had to be aged 18-65, but the results state that participants aged from 11-65. I could not identify which study may be the cause, but while searching I did notice that Abassi et al 2023 appears to not meet the stated inclusion criteria (it was of adolescents aged 15-18).

16. A few studies were excluded post-hoc based on heterogeneity / outlying estimates, without pre-specification or further justification. This is not recommended in standard guidance (e.g. Cochrane handbook) and risks introducing bias. I would at least recommend including these as a sensitivity analysis, and adding stronger theoretical rationale for their exclusion.

Reviewer **2**

Name **Hashimoto, Koshi**

Affiliation **Dokkyo Medical University Saitama Medical Center, Department of Diabetes, Endocrinology and Hematology**

Date **19-Jan-2026**

COI **None**

Overall comment

This manuscript presents a systematic review and network meta-analysis of randomized controlled trials evaluating the effects of different exercise modalities on body composition in women with overweight and obesity. By focusing exclusively on female participants and integrating both direct and indirect evidence across a wide range of exercise interventions, the study addresses an important and clinically relevant question. The methodology is generally sound, the literature search is comprehensive, and the analyses are clearly presented. The findings provide potentially useful information for individualized exercise prescription.

However, several concerns related mainly to interpretation, methodological transparency, and consistency need to be addressed before the manuscript can be considered for publication.

Major concerns

1. SUCRA values reflect the probability of an intervention ranking higher relative to others but do not directly convey effect size magnitude, clinical relevance, or certainty of evidence. In several sections (including the Abstract, Discussion, and Conclusion), SUCRA-based rankings are interpreted in a rather definitive manner (e.g., “most effective” or “optimal”). These statements should be tempered. The authors are encouraged to acknowledge the limitations of SUCRA explicitly and to frame conclusions more cautiously (e.g., “ranked relatively higher” or “appeared more favorable”).

2. Resistance training (RT) is described as having weak effects on body fat percentage. While the primary analysis supports this, the substantial heterogeneity of RT protocols and limited power in some comparisons warrant a more nuanced interpretation. Moreover, RT ranked relatively high for lean body mass in SUCRA analyses. The Discussion should better balance these findings and avoid overly negative conclusions regarding RT as a standalone intervention.

3. Although definitions of exercise intensity are provided, variability in reporting and implementation across primary studies may have resulted in misclassification, particularly between RT and combined AE+RT interventions. The potential impact of such misclassification on network estimates and rankings should be discussed more explicitly, and any relevant sensitivity analyses should be reported or acknowledged.

4. The subgroup analysis by publication year is interesting but appears exploratory. The manuscript currently interprets these findings rather strongly, particularly in the Abstract, suggesting that the “optimal” exercise modality has changed over time. This interpretation risks overstating the findings, as differences may reflect changes in study design, intervention protocols, or populations rather than true temporal shifts in efficacy. These results should be clearly framed as hypothesis-generating.

5. According to the CINeMA assessment, the certainty of evidence for many comparisons is moderate to low. The strength of the conclusions and clinical implications should be aligned more closely with this level of certainty, particularly in the concluding statements.

6. Restricting the analysis to women is a key strength, but the rationale for this decision could be further elaborated, particularly with respect to sex-specific physiology and exercise responses. In addition, the authors should clarify the extent to which their findings can (or cannot) be generalized beyond female populations.

Minor concerns

7. There is an inconsistency between the Methods section (which states that participants are aged 18–65 years) and the Results section, which reports participant ages ranging from 11 to 65 years. This discrepancy requires clarification.

8. The use of BMI and body fat percentage as alternative inclusion criteria should be discussed in terms of potential implications for transitivity.

9. Greater discussion of heterogeneity in body fat measurement methods (e.g., BIA vs DEXA) and their potential influence on effect estimates would strengthen the manuscript.

VERSION 1 - AUTHOR RESPONSE

Reviewer 1 (Statistical Reviewer): Dr Kyle Dack

1. Exclusion of outlier studies not pre-specified

Response:

We thank Reviewer 1 for highlighting this.

Response:

We revised the Methods to state:

We conducted sensitivity analysis to explore impact of extreme effect sizes. Outlier exclusion was not pre-specified; we have now reincluded these studies in sensitivity analyses and report results.

2. Overweight vs obesity are clinically distinct—justification for pooling

Response:

We expanded Introduction and Discussion to explain:

These categories share biological mechanisms but differ in severity.

Prior exercise NMAs often pool overweight and obesity populations.

We treat BMI/BFP as potential effect modifiers and now discuss this as a limitation.

3. Inclusion of Abassi et al. (adolescents 15–18)

Response:

Thank you for identifying this. This study has now been removed from the dataset.

We updated the relevant sections including:

- Study selection flow diagram
- Characteristics table
- All analyses and meta-estimates
- Age range in Results is now corrected to 18–65 years.

4. Abstract should list all databases and ROB tool

Response: We rewrote the abstract and explicitly specified the databases used.

5. Clarify overweight/obesity classification and expand on BMI ranges

Response:

We revised population section:

Added BMI upper limit for overweight (25–29.9 kg/m²)

We have also clarified rationale for region-specific cutoffs and added WHO citation

Population (P): Participants were classified as overweight or obese according to regional BMI cutoffs when available. For European populations, overweight was defined as BMI (25.0–29.9 kg/m²) and obesity as BMI $\geq$ 30 kg/m². For Asian populations, overweight was defined as BMI $\geq$ 24 kg/m² and obesity as BMI $\geq$ 28 kg/m². For studies conducted in other regions or when regional cutoffs were not specified, the BMI classification criteria of the World Health Organization (overweight: BMI $\geq$ 25 kg/m²; obesity: BMI $\geq$ 30 kg/m²) were applied.

6–12. Improvements to PICOS structure, exercise classification, outcomes hierarchy, handling of multi-arm trials, transitivity, intervention duration heterogeneity, and missing reference

Response:

- These sections were comprehensively strengthened. Specifically:
- PICOS moved to beginning of Methods
- Added full explanation of exercise category development
- Added explicit handling of multi-arm trials using methods accounting for within-study correlation
- Discussed BMI/BFP transitivity and heterogeneity in detail
- Added missing citation
- Added discussion on effect of intervention duration

All recommended changes implemented.

13–16. PRISMA flow issues, incorrect stats, age discrepancy, outlier removal

Response:

We updated the flowchart and detailed the reasons for study exclusion. The automated tool had removed an invalid file labeled “Actrn,” and additional records were excluded based on predefined eligibility criteria. We also provided a complete rationale and sensitivity analyses for handling outliers.

Reviewer 2: Dr Koshi Hashimoto

1. Overinterpretation of SUCRA

Response:

We agree and softened all SUCRA-based statements.

Terms such as “optimal,” “most effective,” have been revised to: “ranked higher”, “had a greater probability of effectiveness”, “appeared more favourable”

The manuscript now aligns with CINeMA certainty ratings.

2. Resistance training interpretation is too negative

Response:

We revised discussion to reflect:

We have updated the results section and revised the manuscript to address and remove overly negative wording.

3. Variability and possible misclassification between RT and AE+RT

Response:

We elaborated on:

- Differences in reporting between studies
- Risk of misclassification
- Impact on network structure
- This is now acknowledged explicitly as a limitation.

4. Subgroup analysis overinterpreted

Response:

We have updated the subgroup analysis results and clarified that these findings are exploratory in nature.

5. CINeMA indicates moderate–low certainty—conclusions should reflect this

Response:

We revised Abstract, Discussion, and Conclusion to temper language and align with evidence certainty.

6. Rationale for restricting to women should be expanded

Response:

We added justification based on:

We have expanded the rationale for restricting the analysis to women in the discussion section. Specifically, we now clarify that this decision was made to reduce clinical heterogeneity and to better satisfy the transitivity assumption underlying network meta-analysis. We further elaborate on well-established sex-specific physiological differences—including differences in body fat distribution, hormonal milieu, substrate utilization, and muscle protein synthesis—that may act as effect modifiers in exercise-induced body composition adaptations. In addition, we now explicitly discuss the implications of this restriction for generalizability and emphasize that the findings should not be directly extrapolated to male populations.

7–9. Age discrepancy, BMI/BFP alternative criteria, heterogeneity in measurement methods

Response:

All addressed:

Age range corrected post-removal of adolescent study

We have addressed the alternative inclusion criteria for BMI and body fat percentage in the Discussion section and have added the newly updated subgroup results together with the relevant interpretive discussion.
